# Mesenchymal Stromal Cell Secretome: Influencing Therapeutic Potential by Cellular Pre-conditioning

**DOI:** 10.3389/fimmu.2018.02837

**Published:** 2018-12-04

**Authors:** Joana R. Ferreira, Graciosa Q. Teixeira, Susana G. Santos, Mário A. Barbosa, Graça Almeida-Porada, Raquel M. Gonçalves

**Affiliations:** ^1^Instituto de Investigação e Inovação em Saúde (i3S), Universidade do Porto, Porto, Portugal; ^2^Instituto de Engenharia Biomédica, Universidade do Porto, Porto, Portugal; ^3^Instituto de Ciências Biomédicas Abel Salazar, Universidade do Porto, Porto, Portugal; ^4^Wake Forest Institute for Regenerative Medicine, Winston-Salem, NC, United States

**Keywords:** MSCs (Mesenchymal Stromal Cells), pre-conditioning, regeneration, immunomodulation, therapeutic potential, secretome

## Abstract

Mesenchymal stromal cells (MSCs) are self-renewing, culture-expandable adult stem cells that have been isolated from a variety of tissues, and possess multipotent differentiation capacity, immunomodulatory properties, and are relatively non-immunogenic. Due to this unique set of characteristics, these cells have attracted great interest in the field of regenerative medicine and have been shown to possess pronounced therapeutic potential in many different pathologies. MSCs' mode of action involves a strong paracrine component resulting from the high levels of bioactive molecules they secrete in response to the local microenvironment. For this reason, MSCs' secretome is currently being explored in several clinical contexts, either using MSC-conditioned media (CM) or purified MSC-derived extracellular vesicles (EVs) to modulate tissue response to a wide array of injuries. Rather than being a constant mixture of molecular factors, MSCs' secretome is known to be dependent on the diverse stimuli present in the microenvironment that MSCs encounter. As such, the composition of the MSCs' secretome can be modulated by preconditioning the MSCs during *in vitro* culture. This manuscript reviews the existent literature on how preconditioning of MSCs affects the therapeutic potential of their secretome, focusing on MSCs' immunomodulatory and regenerative features, thereby providing new insights for the therapeutic use of MSCs' secretome.

## Introduction

**Figure 1:**
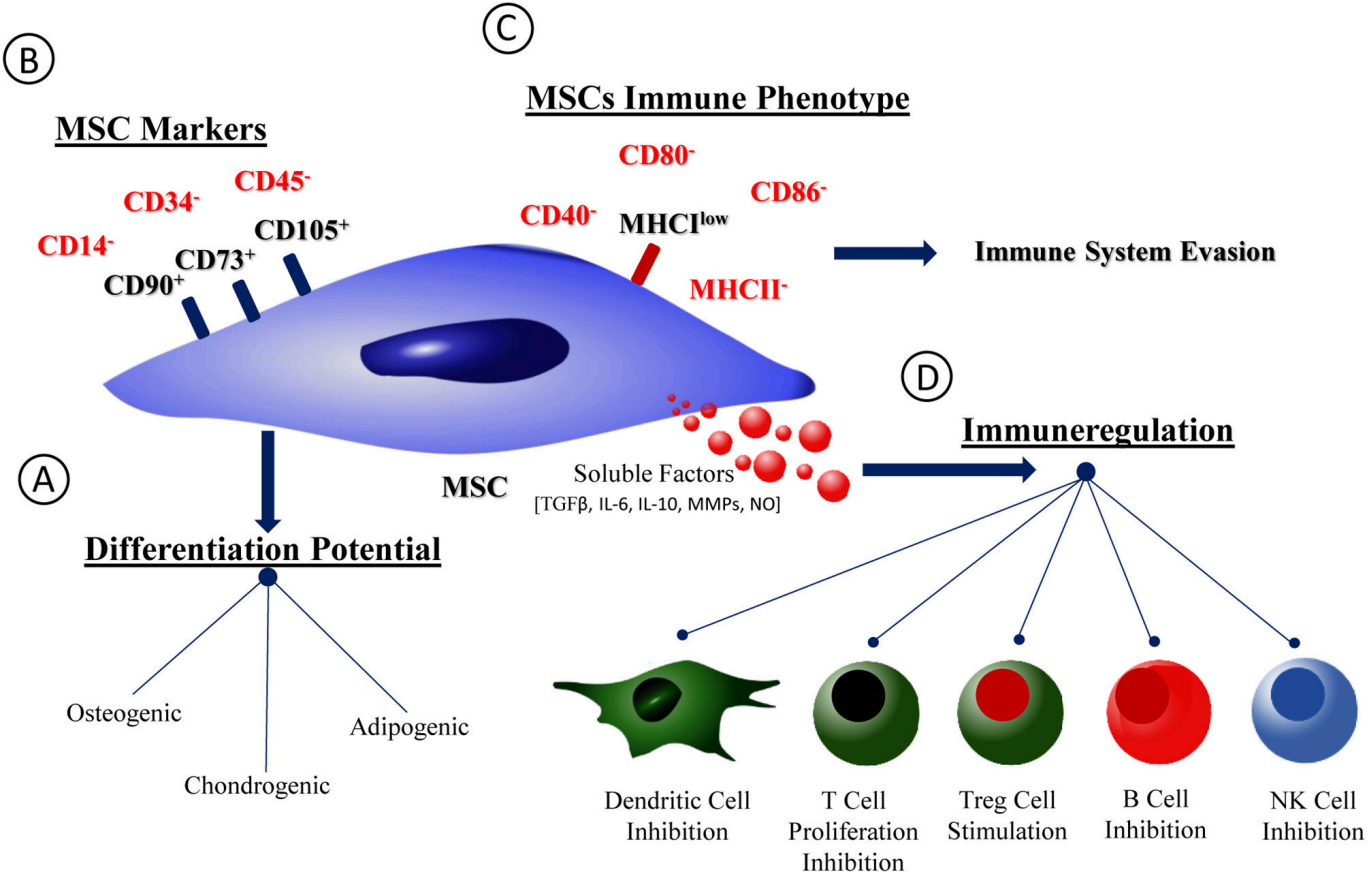
MSCs phenotype, differentiation potential, and immunological properties. Schematic representation of MSCs phenotype and immunological profile. **(A)** MSCs capacity of differentiation into osteogenic, chondrogenic and adipogenic lineages. **(B)** MSCs phenotype accordingly with the International Society for Stem Cell Research (ISSCR). **(C)** MSCs immunological profile. **(D)** Soluble factors families produced by MSCs and profile of interaction with immune cells.

Mesenchymal stromal cells (MSCs), defined by the International Society for Stem Cell Research (ISSCR) as fibroblast-like non-hematopoietic cells, have been explored in recent years due to the clinical promise they hold for tissue repair in regenerative medicine (1, 2). They present a capacity to differentiate into multiple lineages, which was on the basis of the high number of clinical trials using MSCs. By 2015, 493 MSC-based clinical trials were reported (2), a number that greatly increased in the next 2 years, reaching a total of 861 trials in 2018 according to the official database of the US National Institutes of Health. In an effort to address this fast-increasing knowledge base, several reviews have been published to provide a thorough analysis of the evolution of MSC-based clinical trials (3, 4). Perhaps one of the best documented properties of these cells is their ability to promote regeneration in a variety of tissues and to be a major contributor to the positive results achieved in many published papers (5, 6). Indeed, up to 2015 most of the studies with MSCs had focused on their use to treat disorders of the musculoskeletal system, namely in their application to repair bone or cartilage (2). Looking beyond their potential in tissue repair and regeneration, MSCs have also been used extensively for their immunomodulatory properties, for example to treat graft-vs.-host disease (GVHD) (7) and auto-immune diseases such as lupus (8, 9), or Crohn's disease (10). Furthermore, MSCs' clinical potential has been extended to treat myocardial infarction (11, 12), stroke (13), multiple sclerosis (14, 15), liver cirrhosis (16, 17), diabetes (18, 19), lung injuries (20), among others. MSCs are known as relatively immune-inert cells (21), but depending on the context can have immunosuppressive (22–24), or immune-stimulating capacity (25, 26) (see Figure 1).

Despite this great promise, however, their therapeutic benefits are not limited solely to their regenerative abilities. MSCs have also been referred to as trophic “factories” due to the large number of bioactive molecules they secrete in response to the local environment, which then exert paracrine effects upon neighboring cells and tissues (27). Indeed, an increasing number of authors have come to consider these paracrine or trophic properties to be the primary means by which MSCs conduct many of their therapeutic effects (28–30). This conclusion has been furthered by the observation that, in many cases, the number of differentiated cells is far too small to explain the observed response (27). Nevertheless, this paracrine action is known to be influenced by the microenvironment surrounding the cells (31). Therefore, there's a need to understand how *in vitro* culture conditions affect the regenerative and immunomodulatory potential of MSCs' secretome, with the ultimate goal of defining an optimal “cocktail” to precondition MSCs for a given therapeutic application. While the fast pace of research in this field is providing a large amount of data related to MSCs' therapeutic potential, an integrated investigation into how preconditioning can specifically influence the MSC secretome is lacking. To address this deficiency, we performed a comprehensive literature search on the following databases: clinicaltrials.gov, Google Scholar, Scopus, and PubMed, using either direct word-correspondence search or MESH integrated search, with several combinations of the following words: mesenchymal stem cells, hypoxia, inflammatory, pretreatment, preconditioning, stimulation, stimulus, priming, regeneration, immunomodulation, secretome, conditioned medium (CM), paracrine, therapeutic, brain, nervous system, bone, cartilage, kidney, liver, lung, pancreas, cancer, tumor, diabetes, skin, heart, cardiovascular, and intervertebral disc. The compilation of database outputs (~20,000 papers) was analyzed according to the focus of the study and relevance of the results obtained. From these results, articles found within reference lists were also screened and included when relevant to this article, considering the focus on MSCs preconditioning.

## MSCs Secretome: Preclinical and Clinical Evidences of Its Therapeutic Potential

The MSCs-derived cell-free secretome appears to be able to recapitulate many of the properties/effects that have been described for the MSCs themselves. MSCs secretome is enriched in several soluble factors including cytokines, chemokines, immunomodulatory molecules, and growth factors (32). Additionally, paracrine factors produced by cells can be found encapsulated in cell-secreted vesicles. These Extracellular Vesicles (EV) are usually divided according to their size and origin in the cell into exosomes, microvesicles and apoptotic bodies. The smaller nanosized vesicle populations have deserved the most attention. Microvesicles (100–1,000 nm) originate on the plasma membrane, and exosomes (30–120 nm) that are formed in the multivesicular endosomes, have overlapping size ranges and when their separation cannot be completely ascertained are collectively designated EV (33, 34). EV content is thought to mimic that of the cells (35). The exact composition of MSCs' secretome has been investigated to identify the key molecules responsible for MSCs therapeutic potential, with the final goal being the substitution of a cell-free product to achieve the desired therapeutic effect (see Table 1) (32, 36–38, 40–43). Pro-regenerative effects of MSCs secretome have been observed in many different systems, acting by modulating the immune system (44), inhibiting cell death and fibrosis (45, 46), stimulating vascularization (44), promoting tissue remodeling, and recruiting other cells (47).

**Table 1:** Main factors detected in the MSCs secretome.

**Factors**	**References**
BMP	(36)
CCL5/RANTES	(37, 38)
EGF	(38)
FGF	(38)
G-CSF	(39)
GM-CSF	(39)
HGF	(40, 41)
ICAM	(37)
IDO	(37)
IGF	(38, 40–43)
IL-10	(37)
IL-6	(38, 39, 42)
IL-8	(39, 42)
LIF	(42)
MCP-1	(38, 39, 42)
MMP-1	(36)
MMP-2	(36)
MMP-3	(36)
MMP-7	(36)
PDGF	(38)
PGE2	(37)
TGF-β	(37–39, 41)
TIMP-1	(36, 42)
TIMP-2	(36, 42)
VEGF	(38, 40–43)

### Preclinical Evidence

Preclinical evidence of the regenerative potential of MSCs secretome will be briefly described. ASC (adipose tissue-derived MSCs)-CM was able to regenerate/repair mandible lesions in rabbits. In the ASC-CM obtained from 24 h culture in serum-free medium under hypoxic conditions, the authors detected 43 angiogenic factors, 11 of which also appeared to be involved in bone regeneration: IGF-1, TGF-β1, VEGF, Angiogenin, IL-6, PDGF-BB, basic FGF (bFGF), EGF, RANTES, MCP-1, and MCP3 (38). This repertoire of secreted factors seemed to be in accordance with the BM-derived MSCs-CM composition reported by other authors (36, 41, 43, 48), with the remarkable exception that the BM-derived MSCs-CM also contained HGF (41) and BMP-1 (36). HGF in particular seems to be a key factor in MSCs-mediated reversal of hepatic fibrosis (49). Other studies exploring the effect of locally administered MSCs to degenerated tissues found evidence to support the notion that the soluble factors produced in response to the injury played a decisive role in the observed benefits of MSCs administration (50–52).

In the context of intervertebral disc (IVD) injury, MSCs also seem to act via a paracrine role through crosstalk with IVD cells (53–55). In an *ex-vivo* bovine model of pro-inflammatory/degenerated IVDs, MSCs in co-culture were able to immunomodulate the inflammatory reaction mediated by the nucleus pulposus (NP), even though few cells were found to have actually migrated to the disc (56). Zheng et al. further analyzed MSCs-CM effect on the gene expression of NP-like cells, and found an upregulation of KRT19 and downregulation of MMP12 and MGP (57). As MMP12, KRT19, and MGP have been associated with IVD degeneration, the authors suggested that a healthy NP-like phenotype could be restored by MSCs-CM. In fact, it was further proposed that the MSCs' secretome was stimulating IVD progenitor cells activity (54) and the communication mechanism between MSCs and NP cells was at least partially via secretion of microvesicles (58).

Evidence for the pivotal role of MSCs paracrine activity in injured tissues continues to arise in many different systems and pathologic conditions. In 2007, Dai et al. observed that, in myocardial infarction, using MSCs-CM had a similar, albeit less intense, effect to what had been reported earlier for MSCs *per se*, indicating that at least part of the effect that had been observed following MSCs injection could be attributed to soluble factors (59). In the context of neuronal damage, a local injection of MSCs to the lesion site in a stroke model improved coordinated function, inhibited scar tissue formation and cell apoptosis, and stimulated angiogenesis (60). Despite these marked improvements, no neural differentiation of the transplanted MSCs was observed, reinforcing the key role of their paracrine mode of action. Moreover, it has been established that the presence of BDNF, Glial Cell Line-derived Neurotrophic Factor (GDNF), Nerve Growth Factor (NGF), and IGF in the MSCs secretome is necessary to observe the MSCs-induced neuronal survival and differentiation both *in vivo* and *in vitro* (61). Other models in which MSCs-CM has demonstrated therapeutic efficacy include chronic kidney disease, in which administration of MSCs-CM partially rescued kidney function, mainly by attracting endothelial cells, which led to neo-angiogenesis and stimulated wound closure (62). In this study, the authors concluded that the renal-protective paracrine factors present within the MSCs-CM were likely to be VEGF, HGF, and IGF.

MSCs-derived EV, particularly exosomes, have been increasingly shown to contribute to or even completely replicate the therapeutic effects observed with the use of the entire secretome (63). They were shown to improve cardiac function after a porcine myocardial infarction, reducing infarct size and maintaining the systolic and diastolic performance, as a result of inducing neo-revascularization and modulating the inflammatory response (64). Similarly, hBM-MSCs-derived exosomes injected locally 24 h after an induced focal cerebral ischemia were able to reduce the resulting functional impairments through an increase of angioneurogenesis and the modulation of the peripheral immune response (65). Additionally, the treatment seemed to also induce long-term neuroprotection. Other studies reported that MSCs-derived exosomes could mediate the transfer of the micro RNA (miRNA)-133b to neuronal cells, which induced neurite outgrowth and functional recovery after stroke (66), hinting to the importance of this mechanism in the neuronal protective capacity exhibited by MSCs. These effects were also observed by others in different models of ischemic injury (67, 68), even though their ability to modulate the local inflammatory reaction has not been observed by all (67). In another study, a single administration of MSCs-derived microvesicles inhibited apoptosis and stimulated tubular epithelial cell proliferation, thus protecting animals from acute kidney injury (69). Bruno et al. has demonstrated as well that the treatment of acute kidney injury with MSC-EVs leads to functional improvements and reduced mortality through an inhibition of the apoptotic cascade (70). Moreover, treatment with multiple administrations was shown to be significantly more effective than a single administration of the EVs. In a similar fashion, MSCs-derived exosomes were shown to protect hepatocytes and reduce both hepatic inflammation and collagen deposition (45). Indeed, MSCs-derived vesicles have consistently been reported to play a key role in the paracrine activity of these cells.

### Clinical Trials

While the preclinical evidence showing the regenerative and immunomodulatory potential of the MSCs secretome continues to expand rapidly, the clinical studies revolving around this hypothesis are still scarce. Even so, the few clinical trials performed using the product of the MSCs paracrine activity seem to have already established the safety and feasibility of this method, as none of them reported related adverse effects (71–75). Furthermore, the use of the secretome seemed to be effective in improving the clinical outcomes of the involved patients. In the case of alveolar bone regeneration, conditioned media from commercially available BM-MSCs was administered to 8 patients suffering from severe alveolar bone atrophy and needing bone augmentation (75). These patients received either porous pure beta-tricalcium phosphate (β-TCP) or shell-shaped atelocollagen sponge (ACS) scaffold grafts soaked in the CM. After the surgery, minor inflammation of the local tissues was observed with less infiltration of inflammatory cells recorded. The scaffold was gradually replaced by newly formed bone, with no records of bone resorption in any of the cases and early mineralization observed in the augmented bone. IGF-1, VEGF, TGF-β, and HGF were present in the CM, even though molecules typically involved in bone homeostasis, like BMP-2, were not detected by the methodology used.

Clinical trials addressing alopecia (73) and Female Pattern Hair Loss (74) were able to increase hair density after injecting patients not with the MSCs secretome but with a commercially available product containing its protein components. Furthermore, the treatment of one treatment-refractory GVHD patient with MSCs-derived exossomes yielded a pronounced clinical improvement shortly after the administration with a decrease in more than 50% of the IL-1β/INF-γ/TNF-α-producing peripheral blood mononuclear cells (PBMCs) (72). After 4 months, the clinical condition of the patient was still stable, indicating a long-lasting therapeutic effect of the exossomes. Currently, allogeneic MSC-derived exosomes, enriched for miR-124, are reported in a registered clinical trial, directed to stroke patients (http://clinicaltrials.gov).

## The Effect of Pre-conditioning on MSCs Secretome

Although MSCs have an innate potential to induce and/or contribute to regeneration, this potential is now known to be greatly influenced by diverse extrinsic factors such as the tissue source of the MSCs, the health status and age of the MSCs donor, the batch/lot of serum used for the *in vitro* culture of the MSCs, passage number, oxygen concentration, and the presence/absence of a pro-inflammatory environment when the MSCs are infused (76–82). Thus, *in vitro* preconditioning of MSCs with a variety of different factors has been explored to enhance the therapeutic capacity/potential of MSCs, which included: 3D culture (83–85), pharmacological compounds (86–88), inflammatory cytokines (89, 90), and hypoxia (91, 92) (Table 2). Considering MSCs main mechanism of action upon transplantation might be via paracrine signaling, it is somewhat surprising that only a few groups have studied how preconditioning of MSCs affects their secretory profile. This is particularly relevant when the MSCs' secretome may ultimately prove to be an extremely valuable therapeutic tool. The influence of these factors on MSCs' secretome will be reviewed in the following section.

**Table 2:** MSCs preconditioning parameters diversity.

**Pre-conditioning treatment**	**Treatment conditions**	**MSCs Sources**	**References**
Hypoxia	Anoxia to 2% O2, 4−72 h	Placenta, Gingiva, Bone marrow, Adipose tissue, Umbilical Cord Blood	(82, 91–98)
Cytokines, growth factors and hormones	SDF-1, TGF-α, Angiotensin II, INF-γ, TNF-α, Melatonin, Oxytocin 30 min−7 days	Bone marrow, Umbilical blood cord	(89, 90, 99–101)
3D	Aggregates/spheroids, 24 h−4 days	Bone marrow, Adipose tissue, Synovium	(76, 83–85, 102–104)
Pharmacological agents	Atorvastatin, Diazoxide, LPS, Paclitaxel, Curcumin, S1P, Valproate, Lithium, 30 min−48 h	Bone marrow, Adipose tissue, Cell line	(86–88, 101, 105–107)

### Hypoxia

Normoxic oxygen tension, as used for standard cell culture, is the atmospheric pressure (21% O_2_). The term hypoxia, when employed in the context of cell culture is routinely used to refer to oxygen tensions ranging from 0 to 10% (108). The physiological oxygen tension in tissues can vary from 1% in cartilage and bone marrow to 12% in peripheral blood (109). Thus, the 21% O_2_ routinely used for MSCs culture is far higher than the oxygen found physiologically.

In general, hypoxic preconditioning enhances MSCs' regenerative and cytoprotective effects (82, 91–98). Moreover, culturing MSCs in hypoxic conditions has been shown to maintain MSCs' multipotency (110), enhance MSCs proliferation (111), and increase their levels of cytoprotective molecules (98) (Table 3), thereby improving the ability of MSCs to survive in the harsh environment found within injury sites upon transplantation. The beneficial effects of hypoxic culture preconditioning can likely be explained by the fact that MSCs exist *in vivo* in hypoxic environments (131) and hence have the ability to respond to a hypoxic microenvironment through the upregulation of the transcription factor HIF-1α (132). When stabilized due to the lack of oxygen, and dependent upon the increase of phosphorylated Akt and p38 mitogen-activated protein kinase (p38MAPK), this factor binds to the promoter regions of genes responsive to hypoxia, leading to an increase in available glucose (109). As MSCs are capable of switching from aerobic to anaerobic metabolic pathways, they are then able to endure very low oxygen tension values in their microenvironment (133). Therefore, using these culture conditions to precondition MSCs enhances their capacity to survival for longer periods, increases their proliferation rate, and maintains them in an undifferentiated state (109, 131). Small differences, however, in the oxygen tension used to culture MSCs, and in the culture protocol itself, can influence both their ability to differentiate into each of the different mesenchymal lineages (134) and their paracrine production (109). This extreme sensitivity to oxygen tension is an important factor to bear in mind when analyzing results from studies using different preconditioning protocols. The various studies to-date that have used hypoxia as a means of preconditioning MSCs used a concentration up to 2% O2, for a time period of 4–72 h (Table 4). Unfortunately, a high degree of variability exists between the protocols that have been employed, and this must be considered when assessing the MSCs' therapeutic function.

**Table 3:** Dynamics of MSCs secretome composition with cells pre-conditioning.

	**Molecule**	**Preconditioning factors**			**References**
		**Hypoxia**	**Inflammatory stimuli**	**3D culture**
Adhesion	Gal-9		+		(32)
	VCAM-1		+		(112)
	ICAM-1		+		(112)
	ICAM-4		+		(112)
Antioxidation	Catalase	+			(98)
	HO-1	+			(98)
Apoptosis	IL-24			+	(32)
	TRAIL			+	(32)
	CD82			+	(32)
Cell proliferation and differentiation	IGF	+	+	+	(32, 38, 40–43, 46, 85, 101, 113)
	EGF	+		+	(38)
	G-CSF			+	(114)
	TB4	+			(113)
Chemoattraction	CCL2 (MCP-1)	+	+	+	(37, 38, 48, 114)
	CCL5 (RANTES)	+	+		(37, 38, 48, 112)
	CCL7 (MCP-3)	+		+	(38, 48, 114)
	CCL20		+		(112)
	CXCL1		+		(112)
	CXCL2		+		(115)
	CXCL3		+		(112)
	CXCL5		+		(112, 115)
	CXCL6		+		(112, 115)
	CXCL8 (IL-8)	+	+		(42, 47, 112, 115, 116)
	CXCL9		+		(117)
	CXCL10		+		(112, 115, 117)
	CXCL11		+		(112, 115, 117)
	CXCL12 (SDF-1)	+		+	(41, 43, 114)
	CXCR4	+	+	+	(32, 93, 118–120)
	CXCR7	+			(118)
Immunoregulation	TGF-β	+	+	+	(32, 37, 38, 41, 42, 114, 121)
	IDO	+	+		(32, 37, 117, 122–124)
	Factor H		+		(32, 125)
	IL-10	–/+			(37, 126, 127)
	PD-L1		+		(117)
	HLA-G		+		(117)
	IL-1Ra			+	(114)
	PD-L2		+		(117)
	TSG-6			+	(32, 85)
Inflammation	IL-6	+	+	+	(37, 38, 42, 47, 48, 112, 114–116, 119, 128)
	PGE_2_		+	+	(32, 37, 122–124)
	PTX3		+		(115)
	Complement factor B		+		(115)
	Complement factor D		+		(115)
	COX-2		+		(119)
	TNF-α		+		(112)
	IL-23		+		(112)
	IL-16			+	(114)
	IL-7			+	(114)
	IL-11			+	(129)
	IL-2Rα			+	(114, 129)
Metabolism	STC-1			+	(32)
	Cathepsin L1		+		(115)
	Procathepsin B			+	(129)
Migration	MMP-1		+		(36, 115)
	MMP12	+			(130)
Migration Inhibition	PAI-1		+		(115)
	PAI-2		+		(115)
Neuroprotection	BDNF	+			(46, 120)
	GDNF	+			(46)
Osteogenesis	BMP		+	+	(32, 36, 41, 43, 129)
Pluripotency	Oct4	+			(32)
	Rex1	+			(32)
	LIF			+	(42, 114)
Survival	HGF	+	+	+	(32, 40, 41, 85, 98, 113, 130)
	Bcl-2	+		+	(85, 98, 101)
	Akt	+	+		(89, 91, 98, 120)
	HIF-1α	+			(93, 101, 120)
Vascularization	Angiogenin	+		+	(38, 93, 120, 129)
	FGF	+		+	(32, 38, 41–43, 47, 85, 101, 113, 120, 129, 130)
	PDGF	+			(38, 41, 43)
	VEGF	+	+	+	(32, 37, 38, 40–43, 47, 85, 90, 93–95, 101, 113, 114, 120, 121, 129, 130)
	EPO	+			(93, 120)
	EPOR	+			(93)

**Table 4:** Effect of preconditioning on therapeutic potential of MSCs secretome.

**Pre-conditioning treatment**	**Animal**	**Study model**	**MSCs source**	**Treatment conditions**	**Main identified mediators**	**Major conclusions**	**References**
Hypoxia	Rat	*In vitro* ischemic heart	BM	0.5% O_2_ for 12 h	CM	Cytoprotection of ARVCs to hypoxia	(135)
	Mouse	Acute kidney injury	AT	0.5% O_2_ for 48 h	CM	Enhancement of tissue regeneration and renal function. Decrease in levels of IL-1β and IL-6	(130)
	Mouse	Scald skin wound	Placenta	1–5% O_2_ for 72 h	CM (IL-10)	Reduction in scar formation. Inhibition of proliferation and migration of skin fibroblasts	(126)
	Mouse	Excisional skin wound	AT	1/5% O_2_ for 72 h	CM (VEGF, TGF-β1, via TGF-β/SMAD and PI3K/Akt)	Increase in MSCs and skin fibroblasts proliferation. Acceleration of wound closure	(121)
	Mouse	Excisional skin wound	BM	2% O_2_ for 48 h	CM (bFGF, VEGF, IL-6, IL-8)	Enhancemente of proliferation/ migration of fibroblasts, keratinocytes and enthelial cells. Neovascularization and recruitment of macrophages. Acceleration of wound contraction	(47)
Cytokines, growth factors and hormones	Rat	Cutaneous wound	AT	TNF-α (10 ng/mL) for 48 h	CM (IL-6, IL-8)	Acceleration of wound closure. Increase in angiogenesis and infiltration of immune cells into the wound	(116)

HIF-1α activation due to preconditioning MSCs with hypoxia leads to the induction of factors such as VEGF and Angiotensin, promoters of vascularization (136, 137). As neovascularization is a key factor in the regenerative process of damaged tissues, this may account, in itself, for the better therapeutic capacity that has been seen with MSCs pretreated with hypoxia. This hypothesis is supported by a growing number of publications identifying VEGF as a crucial molecule for the observed pro-regenerative effects of MSCs (47, 121, 138, 139). Liu et al. described a direct impact of the hypoxia-preconditioned MSCs treatment on endothelial cell proliferation with a simultaneous reduction in apoptosis (139). In addition, infusion of hypoxia-preconditioned BM-MSCs into the portal vein of rats subject to hepatectomy promoted hepatocyte proliferation and survival and improved serum albumin levels after surgery through a TGF-β dependent mechanism (95). Again, increased production of VEGF was observed. Hypoxia-preconditioning induced MSCs to express higher levels of HIF-1α, and the growth factors GDNF, BDNF, VEGF, Ang-1, and SDF-1, as well as its receptor CXCR4, all of which have been linked to neovascularization, as well as EPO and its receptor EPOR, a neuroprotective and pro-angiogenic molecule (120). Also, when using specifically hypoxia-preconditioned MSCs-derived EVs to treat acute myocardial infarction, authors reported the importance of the increased vascularization in the therapeutic effects. Bian et al. observed that EVs derived from BM-MSCs preconditioned with hypoxia for 72 h were able to significantly improve cardiac function after acute myocardial infarction, mainly through the promotion of angiogenesis (140). Indeed, a comprehensive proteomic analysis of exosomes derived from hypoxia-exposed MSCs showed that these exosomes induce angiogenesis in endothelial cells via the activation of the NFκB pathway (141). However, in another study exosomes derived from hypoxia-preconditioned MSCs contributed to the attenuation of the injury resulting from an ischemia/reperfusion episode via the Wnt signaling pathway (142). Beyond that, hypoxia seems to increase exosome secretion in general (141). Also, in a fat graft model, co-transplantation of exosomes from hypoxia pre-conditioned adipose-derived MSC improved vascularization and graft survival (143) (see Table 5).

**Table 5:** Effect of preconditioning on therapeutic potential of MSCs-derived exosomes.

**Pre-conditioning treatment**	**Animal**	**Study model**	**MSCs source**	**Treatment conditions**	**Main identified mediators**	**Major conclusions**	**References**
Hypoxia	Rat	Acute myocardial infarction	BM	1%O_2_ for 72 h	EVs	Increased angiogenesis and improved cardiac function	(140)
	Rat	I/R cardiac injury	?	?	EVs (miRNA26a)	Attenuation of the injured area and arrythmias	(142)
	Mouse	Acute myocardial infarction	BM	Anoxia + reoxygenation	EVs (miRNA-22)	Reduction of post-infarction fibrosus	(144)
Cytokines, Growth Factors and Hormones	Rat	Kidney ischemia/reperfusion injury	UCB	IFN-γ (100 ng/mL) for 24–48 h	Evs	Loss of cytoprotective effect. Loss of complement factors and lipid binding proteins and gain of tetraspanins, a more complete proteasome complex and MHCI	(145)
Pharmacological agents	Rat	Local cerebral ischemia	Cell line	BYHWD (2,4 g'mL) for 48 h	Evs (VEGF)	Attenuation of ischemic injury by an increase in vascularization	(146)

Nevertheless, other growth factors are also upregulated in response to this stimulus (43, 46, 147) (Table 3), and these factors likely contribute to the specificity of tissue regeneration in a variety of scenarios. An analysis of the hypoxia-preconditioned MSCs' CM used to treat wounds in diabetic rats, revealed higher levels of VEGF, IGF-1, and bFGF (94), while another study reported increased production of VEGF-1α and Bcl-2, with upregulation of HIF-1α, HGF, bFGF, MMP9, and PDGF in MSCs pretreated with hypoxia (101). In agreement with these aforementioned studies, Zhang and colleagues observed that pre-treatment with hypoxia led to increased levels of VEGF, bFGF, and Akt that were implicated in the enhancement of MSCs' anti-oxidative, anti-apoptotic, and pro-angiogenic effects in a rat model of acute kidney injury (96). Additionally, other studies showed that both hypoxia and, to an even greater degree, forced overexpression of *Akt*, upregulated expression of VEGF, bFGF, HGF, IGF, and TB4, molecules associated with tissue repair and regeneration (113). The Akt signaling pathway was also reported to play a role in the enhanced wound healing observed in mice treated with the secretome from hypoxia-preconditioned MSCs (121). The effect of this secretome was related to increased levels of fibronectin, AKT, PI3K, and SMAD2 in the injured tissue; molecules that are all involved in cell proliferation and migration. The hypoxia-preconditioned MSCs secretome was also shown to contain higher levels of VEGF and TGF-β, which led to increased cell proliferation and migration of dermal fibroblasts, via the TGF-β/SMAD2 and PI3K/AKT signaling pathways (121). These results were further validated by Chen et al. who demonstrated that the hypoxia-preconditioned MSCs secretome significantly increased proliferation and migration of keratinocytes, fibroblasts, endothelial cells, and monocytes *in vitro*, and that skin wound contraction was accelerated in an *in vivo* mouse model (47). The secretome produced by hypoxia-preconditioned placenta-derived MSCs was also shown to reduce scar formation and inhibit proliferation and migration of skin fibroblasts *in vitro* (126). In this case, IL-10 was identified as the key player in the process. In agreement with all these results, Lan and colleagues reported increased expression of anti-apoptotic (HGF, Bcl-2), anti-oxidative (catalase, HO-1), and pro-angiogenic (VEGF) factors in hypoxia-treated BM-MSCs infused with the goal of improving the respiratory function of mice suffering from pulmonary fibrosis (98). Chen et al. observed an increase in the MSCs production levels of not only VEGF-A and bFGF, but also IL-6 and IL-8 (molecules involved in the inflammatory response) under hypoxic conditions (47).

The cytoprotective effect of the hypoxia-pretreatment of MSCs, along with changes in metabolism and maintenance of their differentiation potential, have now been repeatedly demonstrated by a variety of authors, despite differences in the hypoxic conditions used (138, 148). From these studies, it has been concluded that hypoxia-preconditioning increases MSCs' survival in harsh environments (148) and enhances their angiogenic capacity, which together boost MSCs' regenerative and immunomodulatory abilities, contributing to the regulation of excessive fibrosis and cell death due to uncontrolled inflammation (96, 98, 101).

### Cytokines, Growth Factors, and Hormones

When considering a significant amount of experimental data regarding MSCs preconditioning with inflammatory cytokines, it is readily apparent that such a stimulus seems to predominantly promote an increase in the production of factors involved in the regulation of the immune response (see Table 3). This includes chemoattraction of most immune cells, modulation of inflammation, and even enhancing migration and homing of transplanted MSCs to sites with higher concentrations of such inflammatory molecules. Their immunoregulatory abilities encompass the inhibition of the complement system activation, the inhibition of NK cells, the guidance of monocyte differentiation toward anti-inflammatory macrophages (M2 phenotype), the suppression of cytotoxic T cell proliferation, and the increase in the numbers of regulatory T cells (149). Many of these outcomes are explained by the large number of chemokines produced by the MSCs that effectively attract numerous immune cells to resolve an inflammatory response (150). Specifically, IL-6, PGE2, and IDO all seem to be major effector molecules in the immunoregulatory effects MSCs mediate (123, 151, 152). The production of this potent triad of immunomodulatory molecules is stimulated by the presence of pro-inflammatory factors such as IL-1β, TNF-α, IFN-γ, and LPS (22, 42, 123, 153, 154), that induce MSCs to adopt an immunomodulatory phenotype and to trigger the production of a cocktail of growth factors. These studies thus collectively indicate the close relationship that exists between inflammation and regeneration. In agreement with this supposition, the therapeutic effects that were observed with TNF-α treated MSCs in a wound closure model, mainly mediated by increased angiogenesis and immune cells infiltration, were observed to be dependent on increased levels of IL-6 and IL-8 (116). Indeed, a recent publication featuring an extensive proteomic analysis of the secretome from BM-MSCs preconditioned with pro-inflammatory factors (IL-1β, IL-6, and TNF-α) clearly demonstrates how a pro-inflammatory stimulus mainly increases MSCs production of proteins involved in inflammation and angiogenesis (155). Moreover, the authors also explore the idea that MSCs role in regulating the proteolytic activity in tissues is key for the regulation of these processes.

Still, the mechanism by which these factors seem to influence MSCs is still largely undefined. There is evidence that MSCs immunomodulatory abilities are mediated by both cell-to-cell contact-derived mechanisms (156–158) and paracrine communication (159–161). Also, some authors believe that MSCs are not naturally immunosuppressive and thus, need licensing at the site of inflammation to become so (162–165). This theory is supported by results demonstrating that molecules such as IFN-γ, TNF-α, or IL-1β are necessary to activate the MSCs immunomodulatory activity (166, 167). One study exploring the effect of the preconditioning with TNF-α on MSCs-derived exosomes demonstrated that the effect the stimulatory effects these vesicles had on human osteoblasts was potentiated through increase of Wnt-3a content in ASC-exosomes (168). Conversely, IFN-γ priming of MSC before EV isolation was reported not to influence the immunomodulatory capacity of exosomes or microparticles, which displayed dose-dependent immunomodulatory effects in inflammatory animal models (169). Additionally, TLRs (Toll-Like Receptor) have also been implicated as important mediators of this activation. Optiz et al. reported that activation of TLR3 and TLR4 lead to the induction of IDO which, in turn, mediated the immunosuppressive actions of the MSCs (170). Activation of TLR-2 was shown to cause an increase in the production of galectin-3 by MSCs and, thus, potentiate their capacity to suppress T-cell activation (171). Nonetheless, contradictory reports have also been published. Liotta et al. demonstrated that TLR3 and TLR4 activation not only increased the production of pro-inflammatory molecules but also reduced their inhibitory effect on the proliferation of T-cells (172). Furthermore, they observed that the activation of these TLRs didn't have any effect on levels of IDO. More recently, along with the demonstration that priming with IFN-γ enhanced MSCs immunosuppressive abilities, mainly through the induction of IDO, it was also shown that TLR3 activation did not affect IDO levels and did not influence the cells immunosuppressive activity (165).

Preconditioning with a myriad of other soluble factors, such as growth factors or hormones, seems to also potentiate MSCs regenerative capacity, mainly by stimulating angiogenesis and inhibiting fibrosis. For example, intracardiac transplantation of SDF-1-preconditioned MSCs increased angiogenesis and reduced fibrosis in the ischemic area of a post-infarct heart (89). The effects observed were attributed to the activation of the Akt signaling pathway, similarly to what was described for hypoxia-preconditioned MSCs. TGF-α-preconditioned MSCs enhanced cardiac function mainly through increased VEGF production via a p38 MAPK-dependent mechanism (90). TNF-α or hypoxia were then combined with the TGF-α during prestimulation, and this led to a further improvement in cardiac function. Once more, VEGF seemed to play a key role in MSCs' mode of action. Preconditioning MSCs with a cocktail of growth-factors (FGF-2, IGF-1, and BMP-2) was also attempted, and this was found to yield protective effects on cardiomyocytes and to improve left ventricular systolic function in a rat myocardial infarction model (173). Another soluble molecule that has been used to precondition MSCs is melatonin, which activates the ERK 1/2 signaling pathway, and consequently enhances cell survival under oxidative stress (100). Thus, melatonin-preconditioned MSCs increased angiogenesis and neurogenesis, reduced infarct size, and improved neurobehavioral outcome in a rat cerebral ischemia model, and once more this seems to have been related to increased VEGF levels (100). Melatonin-preconditioned MSCs also exhibited significantly higher survival rates after intraparenchymal injection in a rat kidney ischemia model (99). This effect was attributed to an upregulation of the enzymes catalase and superoxide dismutase-1 that imbued MSCs with greater antioxidant capacity. Lastly, H_2_O_2_ has been used to precondition MSCs whose exosomes were used to treat and ischemia/reperfusion injury in a rat model (174). The treatment lead to increased vascularization, which led to higher survival rates, and a reduced inflammatory reaction.

### 3-Dimensional (3D) Culture

MSCs culture in a 3-dimensional (3D) environment is another type of preconditioning that aims to more closely mimic the physiological conditions which the cells would see *in vivo*. 3D culture of MSCs, namely as spheroids, induces an increase in the production of factors associated with cell survival and proliferation and vascularization (129, 175) (Table 3). This, in turn, has been shown to increase these cells' immunomodulatory, angiogenic, anti-fibrotic, and anti-apoptotic activities (83, 85, 104, 176). MSCs spheroids decreased neutrophil activity, the levels of the pro-inflammatory molecules TNF-α, IL-1β, CXCL2/MIP-2, PGE2, and plasmin activity in a mouse peritonitis model (83). Other studies demonstrated that 3D culture of MSCs in spheroids seems to stimulate the cells' pro-angiogenic ability, as demonstrated by the implantation of AT-MSCs aggregates leading to an improvement in renal function in a rat model of acute renal ischemia/reperfusion (85). As previously mentioned, the 3D culture of MSCs has also been demonstrated to increase their anti-fibrotic potential. MSCs spheroids were shown to decrease tissue fibrosis in a mouse model of hepatic fibrosis (104). However, in another model system, MSCs' anti-fibrotic activity was shown to be dependent on the dose of MSCs aggregates, with higher density aggregates being unable to regenerate the cartilage in rabbits suffering from full-thickness osteochondral defects (84). Administration of 3D cultures of MSCs aggregates (with more than 125,000 cells) during 24 h increased wound healing rate in mice with full-thickness diabetic wounds. Lower cell doses yielded results similar to those observed in the vehicle-treated mice (76), supporting the notion that the dose used is key.

The spheroid 3D culture creates a microenvironment where inner layers are exposed to much lower levels of oxygen and nutrients, originating an hypoxic environment (177). Although, as a consequence, MSCs express higher levels of molecules associated with apoptosis (32), their immunomodulatory capacity seems secured by the diverse and abundant production of factors involved in inflammation and immune response (83, 114, 177) (Table 3). Accordingly, 3D MSCs-preconditioning was shown to upregulate *TSG-6* expression, as well as *SCT-1* (anti-inflammatory/anti-apoptotic protein), *LIF, IL-24, TRAIL*, and *CXCR4*, a chemokine involved in spheroid-derived-duction of angiogenic factors such as AngiMSCs adhesion to endothelial cells. Wnt signaling cascade seemed to be involved in this effect as the expression of its inhibitor DKK1 was decreased (175). In fact, 3D pre-conditioning of MSCs demonstrated an influence in the production of, not only inflammatory cytokines, but also matrix constituents and degrading enzymes (76, 84, 85), which contributes to their anti-fibrotic capacity. Culturing MSCs as spheroids seems to further enhance their innate pro-angiogenic ability, presumably by increasing their production of angiogenic factors such as Angiogenin, bFGF, VEGF, and HGFa (85, 129). In addition, culturing MSCs in this more physiologically relevant 3D architecture was found to increase their expression of E-cadherin which, by activating the ERK/AKT signaling pathway, was responsible for the higher levels of VEGF production observed (178).

### Pharmacological Agents

Pre-conditioning of MSCs with pharmacological agents may be considered as an alternative option in specific cases. For example, MSCs were pre-conditioned with atorvastatin (a statin associated with the prevention of cardiovascular disease events) (107), oxytocin (hormone) (179), Curcumin (strong anti-oxidant with anti-inflammatory properties) (180), lipopolysaccharide (LPS—endotoxin) (105), and diazoxide (used as a vasodilator in acute hypertension) (86), and their ability to treat myocardial infarction tested. All of the preconditioning protocols enhanced the survival of the transplanted MSCs *in vivo* and led to improved functional recovery and reduced infarct size (86, 105, 107, 179, 180). In general, these effects were due to increased neovascularization and reduced tissue fibrosis. All the studies, except for those using atorvastatin and oxytocin (86, 105, 180) reported a link between the observed effect and increased levels of VEGF, FGF-2, or HGF and activation of the Akt signaling pathway. In concordance, MSCs-derived EVs obtained after preconditioning with Buyang Huanwu Decoction (BYHWD), a drug that has been used for centuries for the treatment of paralysis and stroke, was shown to attenuate brain injury in a rat local cerebral ischemia model by increasing local VEGF levels (146). Atorvastatin seemed to potentiate MSCs' immunomodulatory capacity, decreasing the infiltration of inflammatory cells and the levels of TNF-α and IL-6, via a CXCR4-dependent mechanism, which the authors concluded was the primary mediator of the improvement of functional recovery and reduction of infarct size in a rat stroke model upon MSCs injection (106). Thus, it is becoming clear that MSCs' paracrine response is as dynamic as the microenvironment that surrounds them. Even minor changes in culture conditions or in the microenvironment of the injured tissue can induce dramatically different results (32).

## Conclusion

Extensive evidence now exists to support the benefits of preconditioning of MSCs with respect to improving their capacity to induce regeneration/repair across the wide array of tissues and pathologic conditions in which these cells have been explored. Depending on the preconditioning factors used in the MSCs' culture, different signaling pathways are activated. Understanding how each different stimulus affects MSCs behavior is crucial to validate MSCs preconditioning as a tool to enhance both the safety and the disease-specific therapeutic potential of MSCs for clinical use.

**Figure 2:**
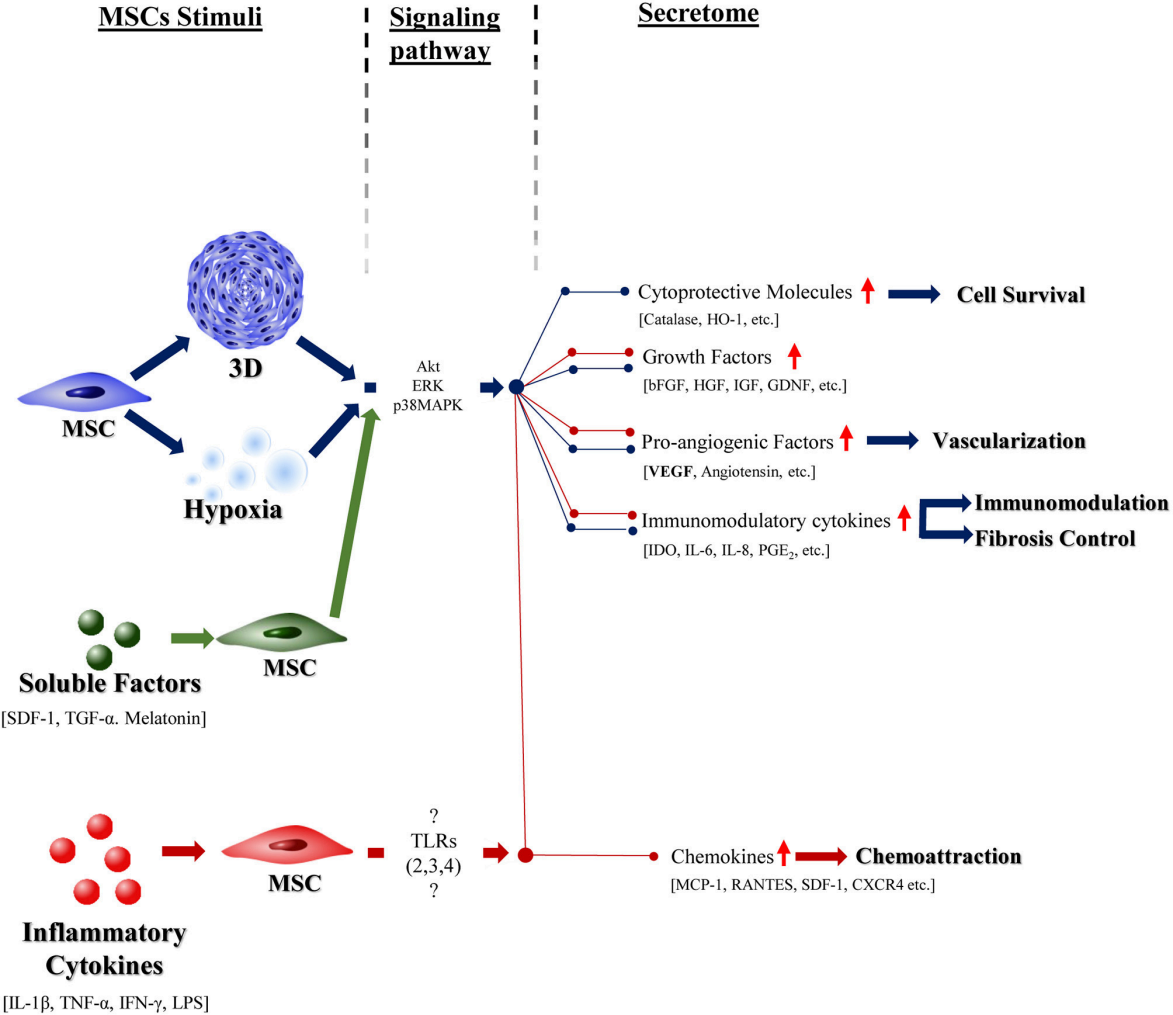
The effect of different preconditioning stimuli in the MSCs response. Schematic representation of known effects of highly studied preconditioning factors—hypoxia (in blue), 3D culture (in blue), specific soluble factors (green), and inflammatory cytokines (red)—in the MSCs response. Blue pathway presents the effect of a hypoxic environment on the cells, which is mediated by specific signaling pathaways (Akt, ERK, p38MAPK) and culminates in the stimulation of the above signaled effects. Tridimensional culture is also represented in blue. MSCs preconditioning with specific soluble factors (SDF-1, TGF-α, and melatonin) seems to stimulate the same signaling pathways as a hypoxic environment and, thus, elicit the same general response from these cells. The use of inflammatory cytokines to influence the MSC response, as represented in red, besides promoting the specific above shown effects, also stimulates the production of factors that seem to be common to all the other preconditioning factors. The pathways that mediate this activity are still to be determined.

Although a great deal of work to comprehend the full mechanisms of MSCs paracrine signaling upon pre-conditioning is needed, some patterns can be recognized (see Figure 2). In summary, pre-conditioning of MSCs with hypoxia, 3D structural organization or soluble factors as SDF-1, or TGF-β seem to activate Akt, ERK, and p38MAPK signaling pathways, that seem to increase the production of cytoprotective molecules (Catalase, HO-1, etc.), pro-regenerative (bFGF, HGF, IGF, etc.) and pro-angiogenic (VEGF) soluble factors and immunomodulatory cytokines (IDO, PGE2, IL6, etc.). On the other hand, priming of MSCs with inflammatory cytokines such as IFN-γ, IL-1β activate TLRs (namely, TLR-2/3/4) on MSCs surface which then increases the production of similar cytoprotective, pro-regenerative, pro-angiogenic and immunomodulatory molecules and further promotes chemokines secretion. Nevertheless, to-date, it has not been possible to identify one single mechanism responsible for this effect.

Moreover, data on the effects that preconditioning of MSCs exerts on the composition and therapeutic potential of their secretome is still lacking. Most studies have focused solely on the effects of hypoxia on the MSCs' secretome content and its therapeutic potential or, in alternative, on the effect that other preconditioning factors could exert on its composition. In most of the studies, there is a lack of evidence on the influence of preconditioning on both the therapeutic effect of the secretome and its composition. Perhaps most importantly, the great majority of studies exploring the clinical utility of the MSCs' secretome have tended to utilize a fairly myopic approach to study its composition, focusing on specific factors of interest in the unique pathological setting being explored, which has contributed to the difficulty in gathering a widely applicable understanding of how the secretome can be fine-tuned for maximal effect in each specific pathology.

Furthermore, due to the wide heterogeneity in MSCs employed in the research community, with respect to their tissue of origin, the health and age of the donor, protocols of cell isolation, culture and preconditioning, as well as the animal model used for testing, it becomes rather difficult to dissect the mechanistic action behind the observed effects on preconditioning of MSCs. Advances in high-throughput techniques and bioinformatic tools, in combination with a database in the area, would help to create a more comprehensive and complete understanding of the way preconditioning can be fine-tuned to increase MSCs' therapeutic utility in the future. This aspect of big data is particularly relevant when the number of studies using MSCs secretome is increasing, accomplished by an expansion of the EVs/exossomes field, in which their secretion by MSCs is being largely explored (181, 182).

Cell therapies as established so far, notwithstanding its great promise, present several obstacles concerning safety, process standardization, and practicality of the procedures needed to deliver viable cells to the hostile microenvironment often present within injured tissues (49, 183, 184). A consensus in the shifting of MSCs' therapeutic potential to their paracrine mechanism of action is being formed within the scientific community, which points to the development of guidelines to refine the experimental settings of the production of MSCs secretome, to establish more standardized protocols among the scientific community and to promote future collaborative work to close the wide gap that has existed for decades between MSCs experimental research and their clinical use.

## Author Contributions

JF and RG conceived the main conceptual ideas and outlined the proof. JF gathered, analyzed, and sorted the revised literature and wrote the manuscript. RG, GT, SS, GA-P, and MB revised and provided critical feedback on the manuscript. RG provided funding.

### Conflict of Interest Statement

The authors declare that the research was conducted in the absence of any commercial or financial relationships that could be construed as a potential conflict of interest.
